# Supplementary material for: Electrochemical reduction of acetonitrile to ethylamine
Source: Nat Commun. 2021 Mar 29;12:1949. doi: 10.1038/s41467-021-22291-0 (PMC8007591; doi:10.1038/s41467-021-22291-0)
Supplement: Supplementary file 1 — Supplementary Information [file 41467_2021_22291_MOESM1_ESM.docx]

**Supplementary Information**

**Electrochemical Acetonitrile Reduction to Ethylamine**

Rong Xia^1,2^, Dong Tian^3^, Shyam Kattel^4^, Bjorn Hasa^1^, Haeun Shin^1^, Xinbin Ma^2*^, Jingguang G. Chen^3*^ and Feng Jiao^1*^

Affiliations:

^1^Center for Catalytic Science and Technology, Department of Chemical and Biomolecular Engineering, University of Delaware, Newark, DE 19716, United States

^2^Key Laboratory for Green Chemical Technology of Ministry of Education, Collaborative Innovation Center of Chemical Science and Engineering, School of Chemical Engineering and Technology, Tianjin University, Tianjin 300072, China

^3^Department of Chemical Engineering, Columbia University, New York, NY 10027, United States

^4^Department of Physics, Florida A&M University, Tallahassee, FL 32307, USA


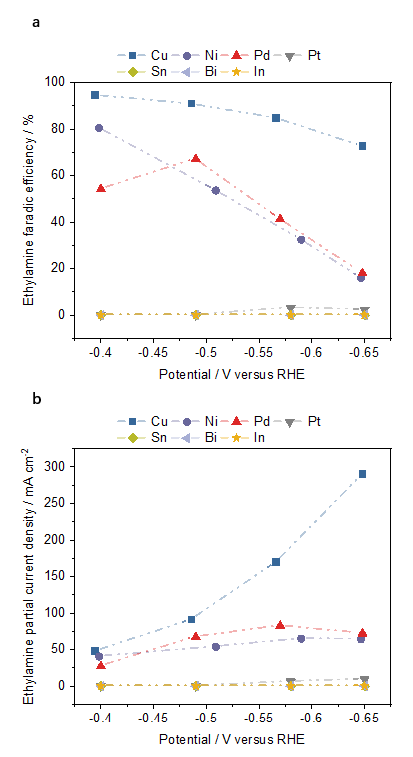


**Supplementary Fig. 1 | Activity of acetonitrile electroreduction over various catalysts. (a)**Ethylamine faradic efficiency and **(b)**ethylamine partial current density plotted independence of applied potentials on various metal catalysts.


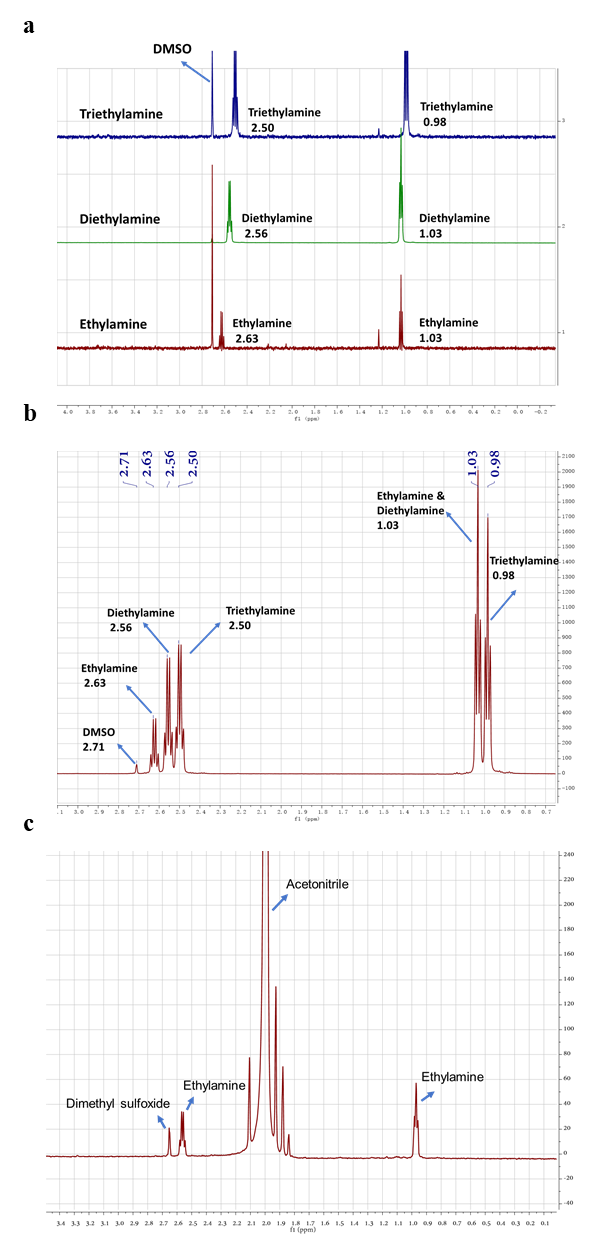


**Supplementary Fig. 2 | NMR spectra.** NMR spectra of (a) ethylamine, diethylamine and triethylamine standard solution, (b) mixture of ethylamine, diethylamine and triethylamine standard solution, (c) typical NMR spectra of effluent electrolyte after the acetonitrile electrochemical reduction.

**Supplementary Fig. 3 | The maximum production rate of ethylamine and H_2_ in the applied potential range of -0.4 V to -0.65 V versus RHE over various catalysts.**

**
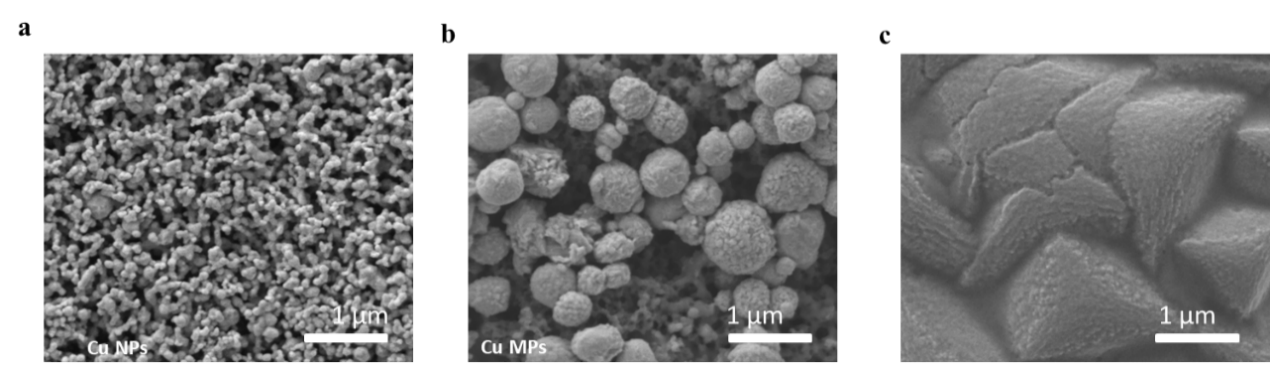
**

**Supplementary Fig. 4| SEM characterization of Cu nanoparticles, Cu microparticles, and oxide-derived Cu on gas diffusion layer.**

Because Cu showed the most promising performance in acetonitrile electroreduction during the catalyst screening, we extended our investigations to three different Cu catalysts, i.e., Cu nanoparticles (25 nm), Cu microparticles (0.5-1.5 µm), and oxide-derived Cu, as shown in the scanning electron microscopy (SEM) images (Supplementary Fig. 4). The catalysts were loaded on a porous carbon paper with a similar loading of 0.5 mg cm^-2^. Fig. 2b shows the partial ethylamine current density on various Cu catalysts in 1 M NaOH electrolyte containing 8 wt.% acetonitrile. The Cu nanoparticles showed a maximum ethylamine partial current density of 557 mA cm^-2^ (a total current density of 1 A cm^-2^ with an ethylamine FE of 55.7%) at -0.76 V, whereas the maximum ethylamine partial current density on Cu microparticles and oxide-derived Cu were 142 mA cm^-2^ and 271 mA cm^-2^, respectively. Moreover, Cu nanoparticles obtained the highest ethylamine FE (96% at -0.29 V) relative to microparticles and OD-Cu (Supplementary Fig. 5). To understand the origin of the high activity of Cu nanoparticles, we compared the intrinsic activities among three Cu catalysts by measuring electrochemical surface areas (ECSA) using the double-layer capacitance method in a batch cell (Supplementary Fig. 6). The ECSA-normalized specific ethylamine partial current densities (Supplementary Fig. 7) for Cu nanoparticles, Cu microparticles, and oxide-derived Cu were 29.8, 26.9, and 22.1 mA cm^-2^, respectively. While Cu nanoparticles have a slightly higher intrinsic activity for acetonitrile electroreduction, the relatively small difference in intrinsic activities indicates that low-coordination sites on the nanostructured catalyst surface have a similar activity as the terrace sites. We, therefore, focused primarily on the Cu nanoparticles as the acetonitrile reduction catalyst in the following studies.

***
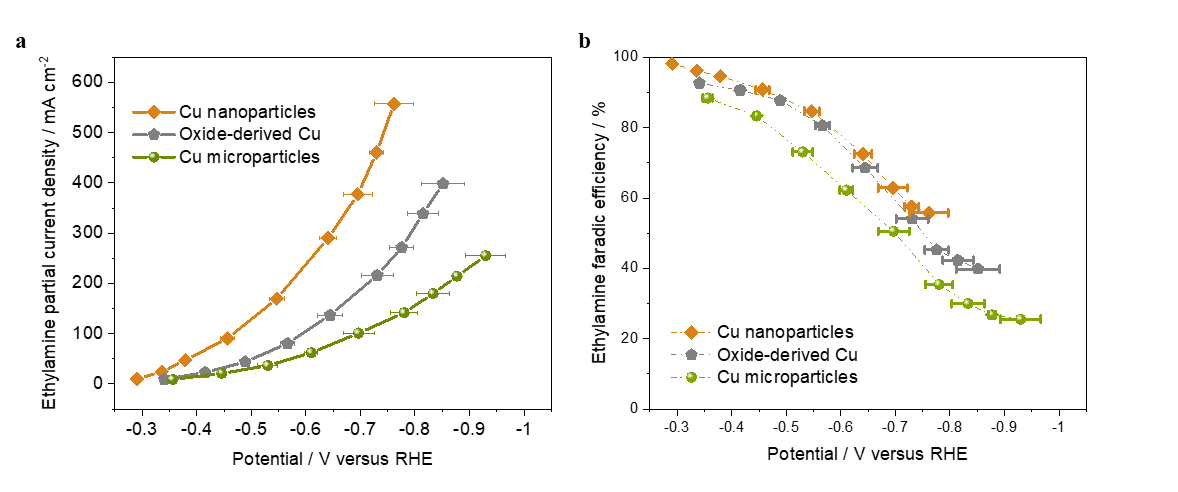
***

**Supplementary Fig. 5| The performance of acetonitrile electroreduction on Cu nanoparticles, Cu microparticles and oxide-derived Cu. (a)** Partial ethylamine current density versus the applied potential for acetonitrile reduction in 8 wt.% acetonitrile in 1 M NaOH on various Cu catalysts. **(b)** Ethylamine FE versus applied potentials on various Cu catalysts in acetonitrile reduction (8 wt.% acetonitrile in 1 M NaOH as electrolyte). Error bars represent the standard deviation from at least three independent measurements.


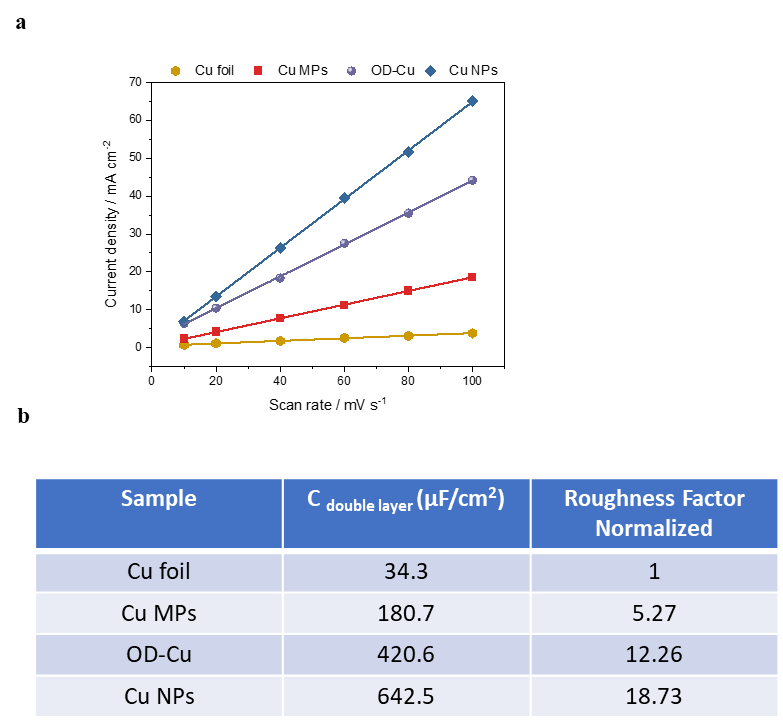


**Supplementary Fig. 6 | Electrochemical chemical surface area (ECSA) analysis of various Cu catalysts.**  **(a)** ECSA measurement of Cu foil, Cu microparticles (Cu MPs), oxide-derived Cu (OD-Cu) and Cu nanoparticles (Cu NPs) in Ar-saturated 0.1 M HClO_4_ electrolyte. **(b)** Measured double layer capacitance of various Cu catalysts and roughness factor relative to Cu foil.

**Supplementary Fig. 7 |** Comparison of ECSA normalized ethylamine partial current density over various Cu catalysts in 8 wt.% acetonitrile in 1 M NaOH. Error bars represent the standard deviation from at least three independent measurements.

***
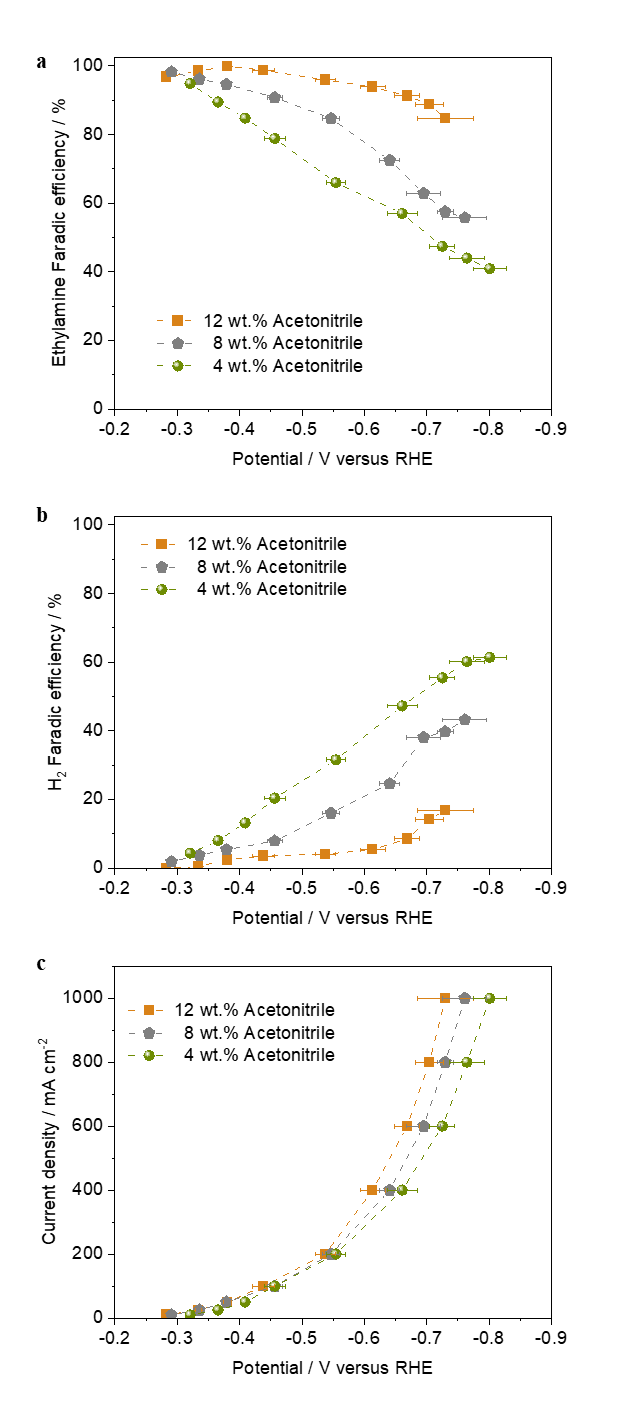
***

**Supplementary Fig. 8 | Comparison of acetonitrile electroreduction performance in different concentration of acetonitrile.** **(a)**Ethylamine FE, **(b)**hydrogen FE, and **(c)** total current density versus potential in 4 wt.%, 8 wt.%, and 12 wt.% acetonitrile in 1 M NaOH. Error bars represent the standard deviation from at least three independent measurements.

**Supplementary Fig. 9 | Acetonitrile order dependence at -0.45 V versus RHE.**

**
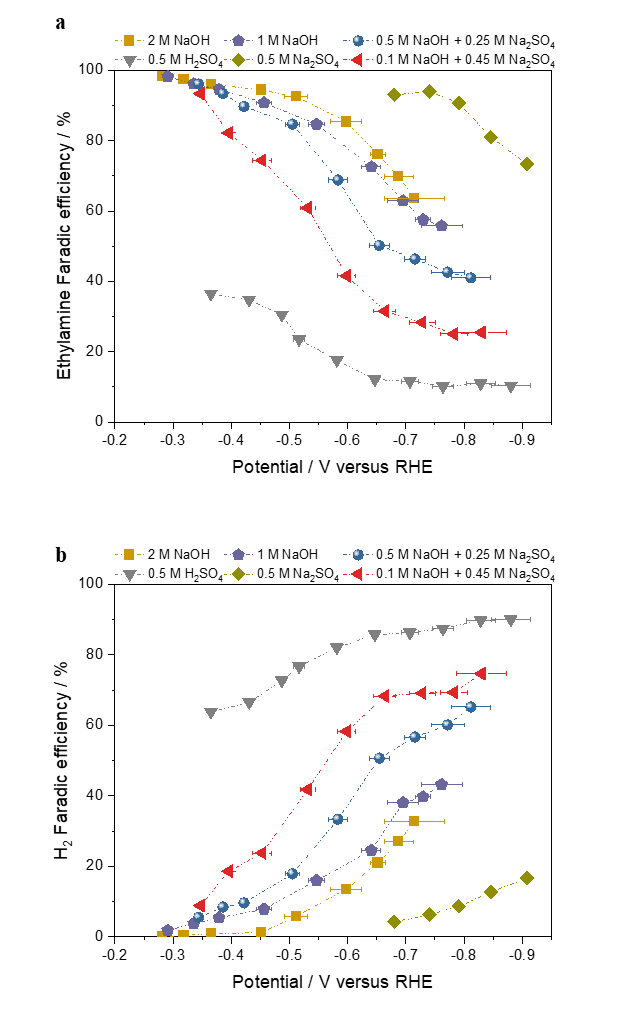
**

**Supplementary Fig. 10 | The pH dependence in acetonitrile electroreduction.** Ethylamine FE **(a)** and hydrogen FE **(b)** plotted against the potential under different electrolyte. Error bars represent the standard deviation from at least three independent measurements.


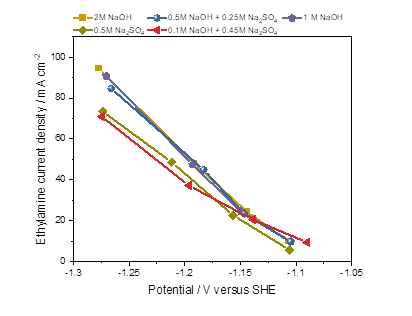


**Supplementary Fig. 11 | Comparison of ethylamine partial current density in electrolyte with different pH on the standard hydrogen electrode (SHE) scale.**


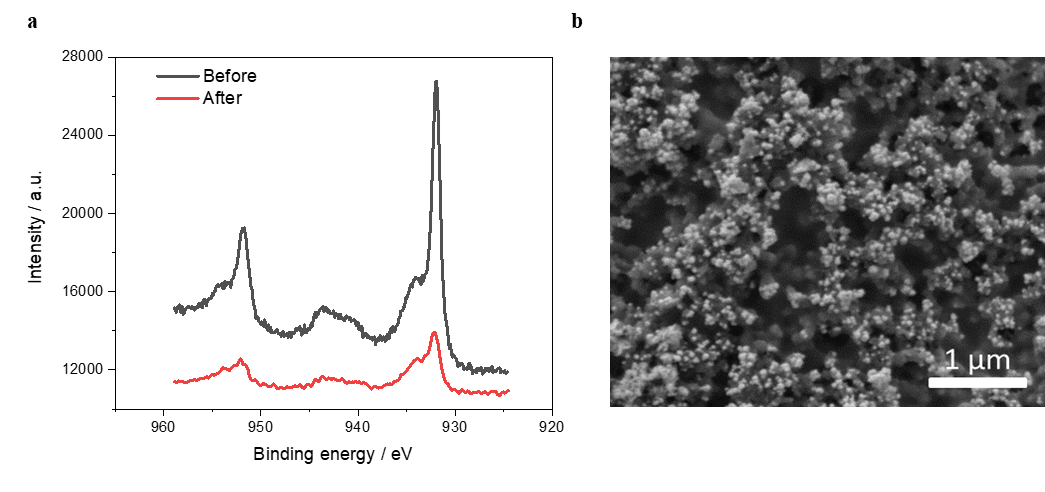


**Supplementary Fig. 12 | Post reaction characterization. (a)**XPS spectrum and **(b)** SEM image of Cu nanoparticles catalysts after the 20-hour stability test in 8 wt.% acetonitrile 1 M NaOH at 100 mA cm^-2^.


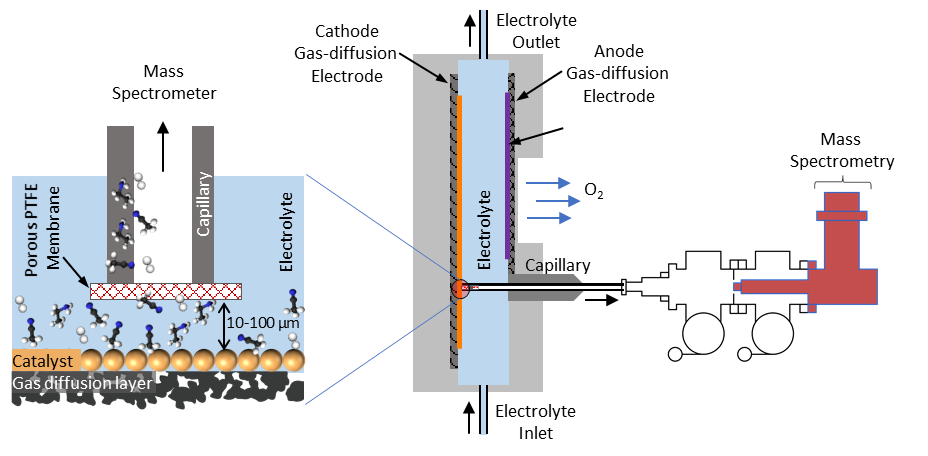


**Supplementary Fig. 13 | Schematic of the flow electrolyzer mass spectrometry.**


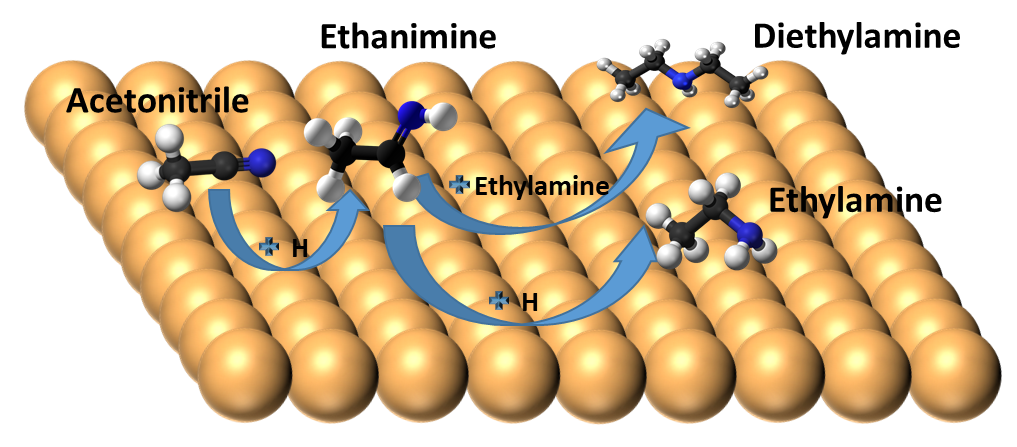


**Supplementary Fig. 14 | Mechanism of acetonitrile reduction to primary amine and secondary amine.**

Previous studies show that due to the strong nucleophilic property of amino group, ethylamine tends to attack the imine (C=N) intermediate in the thermocatalytic acetonitrile hydrogenation, resulting in an equilibrium mixture of ethylamine, diethylamine, and triethylamine. We postulate that the formation of diethylamine in acetonitrile electroreduction may follow a similar mechanism where the ethanimine intermediate reacts with ethylamine, a competing reaction with ethanimine further protonation to ethylamine (Supplementary Fig. 14).


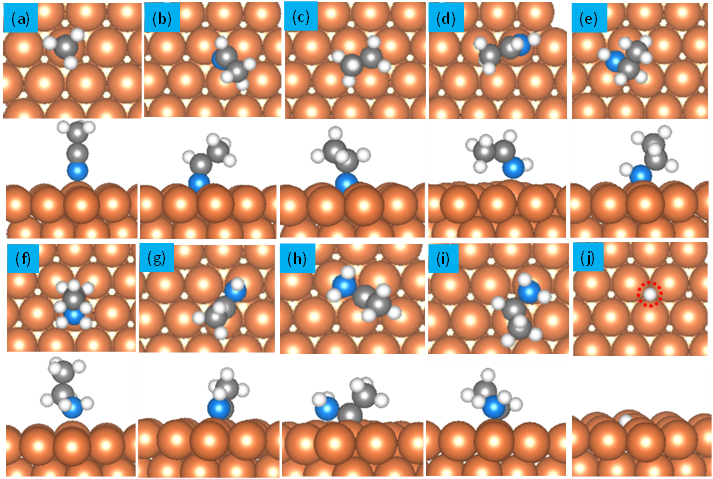


**Supplementary Fig. 15 | DFT optimized geometries on Cu(111) surface.** Top image (side) and bottom image (top) views of **(a)** *CH_3_CN, **(b)** *CH_3_CHN, **(c)***CH_3_CH_2_N, **(d)** *CH_3_CHNH, **(e)** *CH_3_CH_2_NH, **(f)** *CH_3_CH_2_NH_2_, **(g)** *CH_3_CNH, **(h)** *CH_3_CNH_2_, **(i)** *CH_3_CHNH_2_ and **(j)** *H. Cu: Brown, C: gray, N: blue and H: white.

**Supplementary Table 1.** DFT calculated binding energies (BE) of adsorbates on Cu(111), Ni(111), and Pt(111) surfaces.

| Intermediates | BE/eV | | |
| --- | --- | --- | --- |
|  | Cu(111) | Ni(111) | Pt(111) |
| *CH_3_CN | -0.31 | -1.13 | -0.64 |
| *CH_3_CHN | -1.83 | -2.68 | -2.13 |
| *CH_3_CH_2_N | -2.88 | -4.16 | -3.66 |
| *CH_3_CHNH | -0.52 | -0.85 | -1.04 |
| *CH_3_CH_2_NH | -1.88 | -2.47 | -2.27 |
| *CH_3_CH_2_NH_2_ | -0.58 | -0.84 | -1.09 |
| *CH_3_CNH | -1.80 | -2.85 | -3.05 |
| *CH_3_CNH_2_ | -2.45 | -3.82 | -4.28 |
| *CH_3_CHNH_2_ | -0.97 | -1.82 | -2.33 |
| *H | -0.19 | -0.55 | -0.47 |

**Supplementary Table 2**. Various pathways for electrochemical CH_3_CN(g) reduction to CH_3_CH_2_NH_2_(g).

| **Pathway 1** |
| --- |
| CH_3_CN(g) + 4(H^+^ + e^-^) ↔ *CH_3_CN + 4(H^+^ + e^-^) *CH_3_CN + 4(H^+^ + e^-^) ↔ *CH_3_CHN + 3(H^+^ + e^-^) *CH_3_CHN + 3(H^+^ + e^-^) ↔ *CH_3_CH_2_N + 2(H^+^ + e^-^) *CH_3_CH_2_N + 2(H^+^ + e^-^) ↔ *CH_3_CH_2_NH + (H^+^ + e^-^) *CH_3_CH_2_NH + (H^+^ + e^-^) ↔ *CH_3_CH_2_NH_2_ *CH_3_CH_2_NH_2_ ↔ CH_3_CH_2_NH_2_(g) + * |
| **Pathway 2** |
| CH_3_CN(g) + 4(H^+^ + e^-^) ↔ *CH_3_CN + 4(H^+^ + e^-^) *CH_3_CN + 4(H^+^ + e^-^) ↔ *CH_3_CHN + 3(H^+^ + e^-^) *CH_3_CHN + 3(H^+^ + e^-^) ↔ *CH_3_CHNH + 2(H^+^ + e^-^) *CH_3_CHNH + 2(H^+^ + e^-^) ↔ *CH_3_CH_2_NH + (H^+^ + e^-^) *CH_3_CH_2_NH + (H^+^ + e^-^) ↔ *CH_3_CH_2_NH_2_ *CH_3_CH_2_NH_2_ ↔ CH_3_CH_2_NH_2_(g) + * |
| **Pathway 3** |
| CH_3_CN(g) + 4(H^+^ + e^-^) ↔ *CH_3_CN + 4(H^+^ + e^-^) *CH_3_CN + 4(H^+^ + e^-^) ↔ *CH_3_CNH + 3(H^+^ + e^-^) *CH_3_CNH + 3(H^+^ + e^-^) ↔ *CH_3_CNH_2_ + 2(H^+^ + e^-^) *CH_3_CNH_2_ + 2(H^+^ + e^-^) ↔ *CH_3_CHNH_2_ + (H^+^ + e^-^) *CH_3_CHNH_2_ + (H^+^ + e^-^) ↔ *CH_3_CH_2_NH_2_ *CH_3_CH_2_NH_2_ ↔ CH_3_CH_2_NH_2_(g) + * |
| **Pathway 4** |
| CH_3_CN(g) + 4(H^+^ + e^-^) ↔*CH_3_CN + 4(H^+^ + e^-^) *CH_3_CN + 4(H^+^ + e^-^) ↔ *CH_3_CNH + 3(H^+^ + e^-^) *CH_3_CNH + 3(H^+^ + e^-^) ↔ *CH_3_CHNH + 2(H^+^ + e^-^) *CH_3_CHNH + 2(H^+^ + e^-^) ↔ *CH_3_CHNH_2_ + (H^+^ + e^-^) *CH_3_CHNH_2_ + (H^+^ + e^-^) ↔ *CH_3_CH_2_NH_2_ *CH_3_CH_2_NH_2_ ↔ CH_3_CH_2_NH_2_(g) + * |

**Supplementary Fig. 16 |** DFT calculated free energy diagrams for CH_3_CN(g) reduction to CH_3_CH_2_NH_2_(g) along four possible pathways on Cu(111), Ni(111), and Pt(111) at an applied potential U = 0 V.

**Supplementary Fig. 17 |** DFT calculated free energy diagrams for CH_3_CN(g) reduction to CH_3_CH_2_NH_2_(g) along four possible pathways on Cu(111), Ni(111), and Pt(111) at an applied potential U = 0 V.





**Supplementary Fig. 18 | DFT calculated free energy diagrams of HER at an applied potential U = 0 V.**

**Supplementary Table 3**. The highest FE of ethylamine on various metal catalysts and corresponding H_2_ FE in the applied potential range of -0.4 V to -0.65 V versus RHE.

| Catalyst | Ethylamine FE / % | H_2_ FE / % |
| --- | --- | --- |
| Cu | 94.6 | 5.4 |
| Ni | 80.6 | 17.6 |
| Pd | 67.2 | 31.7 |
| Pt | 3.1 | 97.0 |
| Sn | 0.0 | 100.0 |
| In | 0.0 | 100.0 |
| Bi | 0.0 | 100.0 |

**Supplementary Table 4**. The maximum production rate of ethylamine and H_2_ in the applied potential range of -0.4 V to -0.65 V versus RHE over various catalysts.

| Catalyst | Ethylamine production rate / mmol cm^-2^ h^-1^ | H_2_ production rate / mmol cm^-2^ h^-1^ |
| --- | --- | --- |
| Cu | 2.71 | 0.92 |
| Ni | 0.92 | 2.85 |
| Pd | 0.90 | 2.83 |
| Pt | 0.09 | 3.61 |

**Supplementary Table 5**. The ethylamine faradic efficiency and ethylamine partial current density versus applied potentials on various metal catalysts.

| Catalyst | Potential / V versus RHE | Ethylamine Faradic efficiency / % | Ethylamine partial  current density / mA cm^-2^ |
| --- | --- | --- | --- |
| Cu | -0.39 | 94.61 | 47.31 |
|  | -0.49 | 90.79 | 90.79 |
|  | -0.57 | 84.69 | 169.38 |
|  | -0.64 | 72.52 | 290.06 |
| Catalyst | Potential / V versus RHE | Ethylamine Faradic efficiency / % | Ethylamine partial  current density / mA cm^-2^ |
| Ni | -0.41 | 80.56 | 40.28 |
|  | -0.51 | 53.59 | 53.59 |
|  | -0.59 | 32.40 | 64.79 |
|  | -0.66 | 16.00 | 64.00 |
| Catalyst | Potential / V versus RHE | Ethylamine Faradic efficiency / % | Ethylamine partial  current density / mA cm^-2^ |
| Pd | -0.40 | 54.19 | 27.10 |
|  | -0.49 | 67.15 | 67.15 |
|  | -0.57 | 41.23 | 82.47 |
|  | -0.65 | 18.03 | 72.11 |
| Catalyst | Potential / V versus RHE | Ethylamine Faradic efficiency / % | Ethylamine partial  current density / mA cm^-2^ |
| Pt | -0.40 | 0.00 | 0.00 |
|  | -0.49 | 0.00 | 0.00 |
|  | -0.58 | 3.06 | 6.13 |
|  | -0.65 | 2.30 | 9.19 |
| Catalyst | Potential / V versus RHE | Ethylamine Faradic efficiency / % | Ethylamine partial  current density / mA cm^-2^ |
| Sn | -0.40 | 0.00 | 0.00 |
|  | -0.49 | 0.00 | 0.00 |
|  | -0.58 | 0.00 | 0.00 |
|  | -0.65 | 0.00 | 0.00 |
| Catalyst | Potential / V versus RHE | Ethylamine Faradic efficiency / % | Ethylamine partial  current density / mA cm^-2^ |
| Bi | -0.40 | 0.00 | 0.00 |
|  | -0.49 | 0.00 | 0.00 |
|  | -0.58 | 0.00 | 0.00 |
|  | -0.65 | 0.00 | 0.00 |
| Catalyst | Potential / V versus RHE | Ethylamine Faradic efficiency / % | Ethylamine partial  current density / mA cm^-2^ |
| In | -0.40 | 0.00 | 0.00 |
|  | -0.49 | 0.00 | 0.00 |
|  | -0.58 | 0.00 | 0.00 |
|  | -0.65 | 0.00 | 0.00 |

**Supplementary Table 6**.The performance of acetonitrile electroreduction on Cu nanoparticles, Cu microparticles and oxide-derived Cu in 8 wt.% acetonitrile in 1 M NaOH.

| Catalyst | Potential / V versus RHE | Current density / mA cm^-2^ | Ethylamine Faradic efficiency / % | H_2_ faradic efficiency / % |
| --- | --- | --- | --- | --- |
| Cu nanoparticles | -0.29 | 10 | 98.23 | 1.85 |
|  | -0.34 | 25 | 96.15 | 3.77 |
|  | -0.38 | 50 | 94.61 | 5.41 |
|  | -0.46 | 100 | 90.79 | 7.90 |
|  | -0.55 | 200 | 84.69 | 16.00 |
|  | -0.64 | 400 | 72.52 | 24.56 |
|  | -0.70 | 600 | 62.88 | 38.07 |
|  | -0.73 | 800 | 57.53 | 39.73 |
|  | -0.76 | 1000 | 55.73 | 43.20 |
| Catalyst | Potential / V versus RHE | Current density / mA cm^-2^ | Ethylamine Faradic efficiency / % | H_2_ faradic efficiency / % |
| Cu microparticles | -0.36 | 10 | 88.39 | 14.77 |
|  | -0.45 | 25 | 83.34 | 21.10 |
|  | -0.53 | 50 | 73.10 | 30.43 |
|  | -0.61 | 100 | 62.23 | 39.54 |
|  | -0.70 | 200 | 50.51 | 52.88 |
|  | -0.78 | 400 | 35.41 | 66.93 |
|  | -0.83 | 600 | 29.96 | 71.67 |
|  | -0.88 | 800 | 26.78 | 76.28 |
|  | -0.93 | 1000 | 25.53 | 80.61 |
| Catalyst | Potential / V versus RHE | Current density / mA cm^-2^ | Ethylamine Faradic efficiency / % | H_2_ faradic efficiency / % |
| Oxide-derived Cu | -0.34 | 10 | 92.65 | 10.00 |
|  | -0.42 | 25 | 90.66 | 12.98 |
|  | -0.49 | 50 | 87.75 | 17.26 |
|  | -0.57 | 100 | 80.60 | 23.59 |
|  | -0.64 | 200 | 68.54 | 31.77 |
|  | -0.73 | 400 | 54.04 | 47.76 |
|  | -0.78 | 600 | 45.22 | 55.37 |
|  | -0.81 | 800 | 42.32 | 59.57 |
|  | -0.85 | 1000 | 39.82 | 65.08 |

**Supplementary Table 7**. The acetonitrile concentration effect on the electroreduction of acetonitrile.

| Electrolyte | Potential / V versus RHE | Ethylamine Faradic efficiency / % | H_2_ faradic efficiency / % | Current density / mA cm^-2^ |
| --- | --- | --- | --- | --- |
| 12 wt.% Acetonitrile | -0.28 | 96.76 | 0.00 | 10.00 |
|  | -0.33 | 98.53 | 0.42 | 25.00 |
|  | -0.38 | 99.88 | 2.42 | 50.00 |
|  | -0.44 | 98.74 | 3.52 | 100.00 |
|  | -0.54 | 95.89 | 4.10 | 200.00 |
|  | -0.61 | 93.85 | 5.33 | 400.00 |
|  | -0.67 | 91.26 | 8.52 | 600.00 |
|  | -0.70 | 88.73 | 14.14 | 800.00 |
|  | -0.73 | 84.64 | 16.86 | 1000.00 |
| Electrolyte | Potential / V versus RHE | Ethylamine Faradic efficiency / % | H_2_ faradic efficiency / % | Current density / mA cm^-2^ |
| 8 wt.% Acetonitrile | -0.29 | 98.23 | 1.85 | 10.00 |
|  | -0.34 | 96.15 | 3.77 | 25.00 |
|  | -0.38 | 94.61 | 5.41 | 50.00 |
|  | -0.46 | 90.79 | 7.90 | 100.00 |
|  | -0.55 | 84.69 | 16.00 | 200.00 |
|  | -0.64 | 72.52 | 24.56 | 400.00 |
|  | -0.70 | 62.88 | 38.07 | 600.00 |
|  | -0.73 | 57.53 | 39.73 | 800.00 |
|  | -0.76 | 55.73 | 43.20 | 1000.00 |
| Electrolyte | Potential / V versus RHE | Ethylamine Faradic efficiency / % | H_2_ faradic efficiency / % | Current density / mA cm^-2^ |
| 4 wt.% Acetonitrile | -0.32 | 94.85 | 4.31 | 10.00 |
|  | -0.37 | 89.43 | 8.01 | 25.00 |
|  | -0.41 | 84.76 | 13.17 | 50.00 |
|  | -0.46 | 78.85 | 20.37 | 100.00 |
|  | -0.55 | 65.95 | 31.56 | 200.00 |
|  | -0.66 | 56.98 | 47.25 | 400.00 |
|  | -0.73 | 47.38 | 55.45 | 600.00 |
|  | -0.76 | 43.98 | 60.13 | 800.00 |
|  | -0.80 | 40.88 | 61.40 | 1000.00 |

**Supplementary Table 8**. The pH effect on the electroreduction of acetonitrile.

| Electrolyte | Potential / V versus RHE | Current density / mA cm^-2^ | Ethylamine Faradic efficiency / % | H_2_ faradic efficiency / % |
| --- | --- | --- | --- | --- |
| 2 M NaOH | -0.28 | 10 | 98.37 | 0.00 |
|  | -0.32 | 25 | 97.44 | 0.48 |
|  | -0.37 | 50 | 96.05 | 0.82 |
|  | -0.45 | 100 | 94.40 | 1.22 |
|  | -0.51 | 200 | 92.54 | 5.84 |
|  | -0.60 | 400 | 85.33 | 13.52 |
|  | -0.65 | 600 | 76.14 | 21.03 |
|  | -0.69 | 800 | 69.74 | 27.01 |
|  | -0.71 | 1000 | 63.53 | 32.70 |
| Electrolyte | Potential / V versus RHE | Current density / mA cm^-2^ | Ethylamine Faradic efficiency / % | H_2_ faradic efficiency / % |
| 1 M NaOH | -0.29 | 10 | 98.23 | 1.85 |
|  | -0.34 | 25 | 96.15 | 3.77 |
|  | -0.38 | 50 | 94.61 | 5.41 |
|  | -0.46 | 100 | 90.79 | 7.90 |
|  | -0.55 | 200 | 84.69 | 16.00 |
|  | -0.64 | 400 | 72.52 | 24.56 |
|  | -0.70 | 600 | 62.88 | 38.07 |
|  | -0.73 | 800 | 57.53 | 39.73 |
|  | -0.76 | 1000 | 55.73 | 43.20 |
| Electrolyte | Potential / V versus RHE | Current density / mA cm^-2^ | Ethylamine Faradic efficiency / % | H_2_ faradic efficiency / % |
| 0.5 M NaOH + 0.25 M Na_2_SO_4_ | -0.34 | 10 | 96.11 | 5.52 |
|  | -0.39 | 25 | 93.37 | 8.46 |
|  | -0.42 | 50 | 89.71 | 9.64 |
|  | -0.51 | 100 | 84.68 | 17.91 |
|  | -0.58 | 200 | 68.80 | 33.36 |
|  | -0.65 | 400 | 50.20 | 50.69 |
|  | -0.72 | 600 | 46.36 | 56.67 |
|  | -0.77 | 800 | 42.58 | 60.14 |
|  | -0.81 | 1000 | 41.02 | 65.24 |
| Electrolyte | Potential / V versus RHE | Current density / mA cm^-2^ | Ethylamine Faradic efficiency / % | H_2_ faradic efficiency / % |
| 0.5 M H_2_SO_4_ | -0.36 | 5 | 36.48 | 63.85 |
|  | -0.43 | 10 | 34.75 | 66.53 |
|  | -0.49 | 25 | 30.46 | 72.69 |
|  | -0.52 | 50 | 23.50 | 76.71 |
|  | -0.58 | 100 | 17.61 | 82.05 |
|  | -0.65 | 200 | 12.11 | 85.77 |
|  | -0.71 | 400 | 11.61 | 86.29 |
|  | -0.76 | 600 | 10.12 | 87.43 |
|  | -0.83 | 800 | 10.94 | 89.82 |
|  | -0.88 | 1000 | 10.33 | 90.01 |
| Electrolyte | Potential / V versus RHE | Current density / mA cm^-2^ | Ethylamine Faradic efficiency / % | H_2_ faradic efficiency / % |
| 0.5 M Na_2_SO_4_ | -0.68 | 1 | 93.00 | 4.25 |
|  | -0.74 | 5 | 94.00 | 6.35 |
|  | -0.79 | 10 | 90.62 | 8.67 |
|  | -0.85 | 25 | 81.00 | 12.71 |
|  | -0.91 | 50 | 73.35 | 16.70 |
| Electrolyte | Potential / V versus RHE | Current density / mA cm^-2^ | Ethylamine Faradic efficiency / % | H_2_ faradic efficiency / % |
| 0.1 M NaOH + 0.45 M Na_2_SO_4_ | -0.35 | 10 | 93.37 | 8.88 |
|  | -0.40 | 25 | 82.20 | 18.61 |
|  | -0.45 | 50 | 74.39 | 23.79 |
|  | -0.53 | 100 | 60.90 | 41.80 |
|  | -0.60 | 200 | 41.56 | 58.33 |
|  | -0.66 | 400 | 31.55 | 68.31 |
|  | -0.73 | 600 | 28.34 | 69.17 |
|  | -0.78 | 800 | 25.05 | 69.35 |
|  | -0.83 | 1000 | 25.45 | 74.67 |

**Preparation of oxide-derived Cu (OD-Cu).** OD-Cu was reduced from electrodeposited Cu_2_O film reported in a previous study^1^. The Cu_2_O film was deposited on gas diffusion layer through an electrodeposition process. A 2.5 cm^2^ carbon paper was used as substrate and was immersed in the electrolytic bath (0.4 M CuSO_4_ aqueous solution containing 3 M lactic acid, pH=11.5 after adjusted by NaOH). The Ag/AgCl and graphite rod was used as reference electrode and counter electrode, respectively. The electrodeposition was performed in 60^o^C water bath at -0.4 V versus RHE and the metal loading was controlled by monitoring the charge passed during the electrolysis. The as-prepared Cu_2_O film was rinsed with DI water and dried in vacuum overnight. Then it was reduced in 1 M NaOH under -0.4 V for 15 minutes to obtain oxide-derived Cu right before testing the performance.

**Supplementary References**

1 Chang, X., Malkani, A., Yang, X. & Xu, B. Mechanistic Insights into Electroreductive C–C Coupling between CO and Acetaldehyde into Multicarbon Products. *Journal of the American Chemical Society* **142**, 2975-2983, (2020).
